# FABP4 Is an Indispensable Factor for Regulating Cellular Metabolic Functions of the Human Retinal Choroid

**DOI:** 10.3390/bioengineering11060584

**Published:** 2024-06-07

**Authors:** Hiroshi Ohguro, Megumi Watanabe, Tatsuya Sato, Nami Nishikiori, Araya Umetsu, Megumi Higashide, Toshifumi Ogawa, Masato Furuhashi

**Affiliations:** 1 Departments of Ophthalmology, School of Medicine, Sapporo Medical University, S1W17, Chuo-ku, Sapporo 060-8556, Japan; ooguro@sapmed.ac.jp (H.O.); watanabe@sapmed.ac.jp (M.W.); nami076@yahoo.co.jp (N.N.); araya.umetsu@sapmed.ac.jp (A.U.); megumi.h@sapmed.ac.jp (M.H.); 2 Departments of Cardiovascular, Renal and Metabolic Medicine, Sapporo Medical University, S1W17, Chuo-ku, Sapporo 060-8556, Japan; a08m024@yahoo.co.jp (T.O.); furuhasi@sapmed.ac.jp (M.F.); 3 Departments of Cellular Physiology and Signal Transduction, Sapporo Medical University, S1W17, Chuo-ku, Sapporo 060-8556, Japan

**Keywords:** human ocular choroidal fibroblasts (HOCFs), FABP4, mitochondrial respiration, glycolysis, Seahorse bioanalyzer, RNA sequencing, Gene Ontology (GO) enrichment analysis, ingenuity pathway analysis (IPA)

## Abstract

The purpose of the current study was to elucidate the physiological roles of intraocularly present fatty acid-binding protein 4 (FABP4). Using four representative intraocular tissue-derived cell types, including human non-pigmented ciliary epithelium (HNPCE) cells, retinoblastoma (RB) cells, adult retinal pigment epithelial19 (ARPE19) cells and human ocular choroidal fibroblast (HOCF) cells, the intraocular origins of FABP4 were determined by qPCR analysis, and the intracellular functions of FABP4 were investigated by seahorse cellular metabolic measurements and RNA sequencing analysis using a specific inhibitor for FABP4, BMS309403. Among these four different cell types, FABP4 was exclusively expressed in HOCF cells. In HOCF cells, both mitochondrial and glycolytic functions were significantly decreased to trace levels by BMS309403 in a dose-dependent manner. In the RNA sequencing analysis, 67 substantially up-regulated and 94 significantly down-regulated differentially expressed genes (DEGs) were identified in HOCF cells treated with BMS309403 and those not treated with BMS309403. The results of Gene Ontology enrichment analysis and ingenuity pathway analysis (IPA) revealed that the DEGs were most likely involved in G-alpha (i) signaling, cAMP-response element-binding protein (CREB) signaling in neurons, the S100 family signaling pathway, visual phototransduction and adrenergic receptor signaling. Furthermore, upstream analysis using IPA suggested that NKX2-1 (thyroid transcription factor1), HOXA10 (homeobox A10), GATA2 (gata2 protein), and CCAAT enhancer-binding protein A (CEBPA) were upstream regulators and that NKX homeobox-1 (NKX2-1), SFRP1 (Secreted frizzled-related protein 1) and TREM2 (triggering receptor expressed on myeloid cells 2) were causal network master regulators. The findings in this study suggest that intraocularly present FABP4 originates from the ocular choroid and may be a critical regulator for the cellular homeostasis of non-adipocyte HOCF cells.

## 1. Introduction

Among the fatty acid-binding protein (FABP) family coordinating various lipid responses, it is demonstrated that the FABP4 essentially expressed in both adipocytes and macrophages can be secreted into bodily fluids and is physiologically involved in various cellular functions, including fatty acid (FA)-mediated roles [1,2]. FABP4 has also been recognized as an important pathological key factor in various systemic diseases, including obesity, diabetes mellitus (DM), hypertension (HT), hyperlipidemia (HL), atherosclerosis, renal disease, metabolic dysfunction, heart events and malignancy [3,4,5,6,7,8].

In the field of ophthalmology, we have comprehensively demonstrated that FABP4 is also present in vitreous fluid and that levels of FABP4 are significantly increased in patients with retinal vascular diseases (RVDs) such as proliferative diabetic retinopathy (PDR) [9] and retinal vein occlusion (RVO) [10], which are representative vision-threatening retinal complications of DM, HT and HL [11,12,13,14]. In addition, we also showed that levels of total fatty acids (FAs) in vitreous fluids are significantly elevated in RVD patients compared with levels in non-RVD patients [15]. Interestingly, levels of FAs in vitreous fluids were significantly correlated with levels of vitreous FABP4, whereas levels of FAs, vascular endothelial growth factor (VEGFA) and FABP4 in vitreous fluids were not correlated with their plasma levels [15]. Based on these collective results, it has been speculated that FABP4 is independently involved in the molecular pathogenesis of these RVDs with VEGFA, which is recognized as the most critical pathogenic factor for RVDs, and thus, we have suggested that FABP4 is a rationally promising therapeutic target other than VEGFA. Since there were still limited information on the extent of the involvement of FABP4 in RVD etiology and several unsolved questions, including the origin of the intraocular FABP4 despite no fatty tissues being intraocularly present, we have assessed the expression sites of the FABP family in the intraocular tissues in human retinas, wild-type (WT) rat and mouse retinas and in those in DR and retinitis pigmentosa (RP) models by immunolabeling. In these projects, we found that FABP3, 4, 7 and 8 are expressed in the human retina, but only FABP4 and 12 are expressed in rodent retinas, and the immunolabeling patterns of FABP4 for WT, DR and RP rodent retinas are different [15]. Furthermore, amplitudes of electroretinograms (ERGs) of FABP4-deficient (Fabp4^−/−^) mice were significantly increased in comparison with those of WT mice [15]. These findings strongly suggest that intraocular FABP4 plays roles in physiological functions such as phototransduction. However, the specific origins of FABP4 production and its molecular–functional relationships in the intraocular tissues remain unclear.

Therefore, here, we assessed the intraocular origin of FABP4 production in four representative intraocular tissue-derived cell types, including human non-pigmented ciliary epithelium (HNPCE) cells, retinoblastoma (RB) cells, adult human retinal pigment epithelial-19 (ARPE19) cells and human ocular choroidal fibroblast (HOCF) cells. Subsequently, the cellular metabolic functions of cells expressing FABP4 were analyzed by administering the specific FABP4 inhibitor BMS309403 using the Seahorse bioanalyzer in a real-time manner, and gene expression patterns were assessed using RNA sequencing analysis.

## 2. Materials and Methods

### 2.1. Two-Dimensional (2D) Culture of HOCF Cells, HNPCE Cells, RB Cells and ARPE19 Cells

All experimental protocols using human-derived cells were followed in compliance with the tenets of the Declaration of Helsinki after approval by the internal review board of Sapporo Medical University. Human ocular choroidal fibroblast cells (HOCF cells, Cat. #6620, Science Research Laboratories, Inc., Carlsbad, CA, USA) were purchased and cultured in 2D culture dishes (150 mm) until the reached 90% confluence at 37 °C in the recommended fibroblast medium (FM, Cat. #2301, Science Research Laboratories, Inc., Carlsbad, CA, USA). HNPCE (Cat. #6580, Science Research Laboratories, Inc., Carlsbad, CA, USA), RB (Cat. #HTB-169™, ATCC, Manassas, VA, USA) and ARPE19 (#CRL-2302™, ATCC, Manassas, VA, USA) were separately cultured in 150 mm planar culture dishes until they reached 90% confluence at 37 °C in growth medium composed of high-glucose DMEM containing 10% FBS, 1% L-glutamine and 1% antibiotic-antimycotic. These cells were maintained by changing the medium every other day under standard humid normoxia conditions (37 °C, 5% CO_2_). Documents of cell specification for these cells are included in the Supplemental Materials.

To achieve HOCF cell culture under hypoxic conditions, we used a CO_2_ cell culture incubator (Mini-cell35, WAKENBTECH. Co., Kyoto, Japan) equipped with both CO_2_ and O_2_ sensors, by which a 5% CO_2_ and 1% O_2_ hypoxic condition was precisely adjusted and maintained at 37 °C.

To inhibit FABP4 activity in HOCF cells, a specific FABP4 inhibitor, BMS309430 (Cat. #10010206, Cayman Chemical, Ann Abor, MI, USA), ranging from 2.0 to 25.0 μM was administered.

### 2.2. Measurement of Seahorse Cellular Metabolic Functions

Under different concentrations of the FABP4 inhibitor BMS309430, ranging from 0 to 25.0 μM, 2D cultured HOCF cells were subjected to analyses by a Seahorse XFe96 Bioanalyzer (Agilent Technologies., Santa Clara, CA, USA) to measure the oxygen consumption rates (OCRs) and the extracellular acidification rates (ECARs) according to the manufacturer’s instructions, as described previously [16].

### 2.3. RNA Sequencing, Gene Function and Analysis of Pathways

Total RNA was obtained from 2D confluent cells of HOCF cells that were untreated or treated with 5 μM BMS309430 for 24 h in a 150 mm dish using an RNeasy mini kit (Qiagen, Valencia, CA, USA), and following, RNA extraction and next-generation sequencing were performed, as described recently [17]. Obtained sequence data were filtered using FastQC software (version 0.11.7), checked for quality control by an Agilent 2100 Bioanalyzer (Agilent, Santa Clara, CA, USA) and Trimmomatic (version 0.38) and mapped to the reference genome sequence (GRCh38) using HISAT2-2.1.1 tools software [18]. The read counting for each respective gene and the statistical analysis were processed using featureCounts (version 1.6.3) and DESeq2 (version 1.24.0), respectively. Differentially expressed genes (DEGs) were determined as genes with fold-changes ≧ 2.0 and false discovery rate (FDR)-adjusted *p*-values < 0.05 and *q* < 0.08 between groups.

Ingenuity pathway analysis (IPA, Qiagen, https://www.qiagenbioinformatics.com/products/, accessed on 9 February 2024) [19] was used for further analysis to predict various pathways by uploading the Excel file of significant up-regulated and down-regulated DEGs for IPA core analyses. Enrichment of particular genes in networks in the IPA was evaluated using Fisher’s exact test. In addition, the IPA software orders the top functions related with each network based on the enrichment scores (z-score) and predicts possible upstream regulators and causal network regulators, as shown in recent studies [19,20,21].

### 2.4. Other Analytical Methods

The concentrations of FABP4 of cell culture medium were measured using commercially available enzyme-linked immunosorbent assay kits for FABP4 (Biovendor R&D, Modrice, Czech Republic). Real-time PCR was carried out essentially as previously reported [22] using predesigned primers (Supplemental Table S1). The expression of each respective gene was normalized by using the expression of a housekeeping gene, 36B4 (Rplp0). As experimental data, the arithmetic mean ± the standard error of the mean (SEM) was used in conjugation with statistical analyses, essentially as described in our previous reports [22].

## 3. Results

### 3.1. HOCF Cells Are the Primary Origin of Intraocularly Produced FABP4 among Cells Constituting the Intraocular Tissues

**Figure 1:**
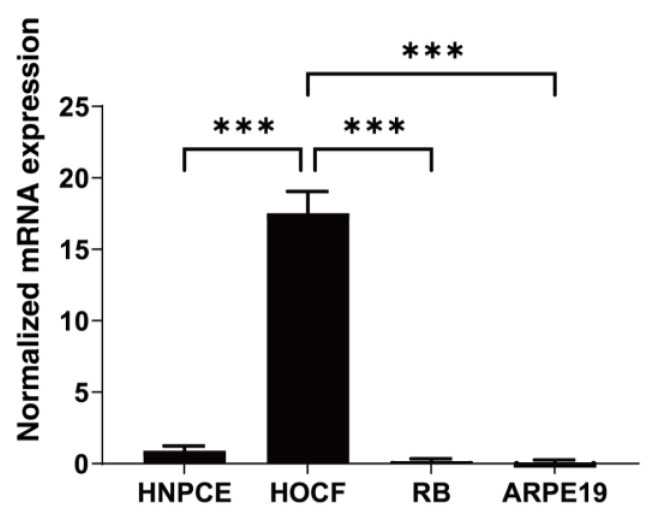
mRNA expression of FABP4 in HNPCE, RB, ARPE19, HOCF and RSC cells. Two-dimensional cultured HNPCE, Rb, ARPE19 and HOCF cells were subjected to qPCR analysis, and the mRNA expression of FABP4 was estimated. Experiments were repeated three times using freshly prepared 2D HOCF cells (n = 3 each) in each. *** *p* < 0.005.

Initially, to determine the intraocular origin of FABP4, the mRNA expression of FABP4 in four representative intraocular tissue-derived cell types, including HNPCE cells, RB cells, ARPE19 cells and HOCF cells, was determined by qPCR, with which human orbital adipocyte was used as the control cells. As shown in Figure 1, FABP4 was only significantly expressed in HOCF cells among the four types of intraocular tissue-derived cells, while gene expression of FABP4 was negligible in the other cells (Figure 1). In addition, approximately 50~220 pg/mL of FABP4 was detected in the 2 mL of culture medium obtained from confluent HOCF cells in the wells of 12-well culture plates from a commercially available ELISA kit for FABP4. Collectively, these results suggested that HOCF cells are at least one of the primary origins of FABP4 production in cells constituting the intraocular tissues.

### 3.2. Effects of FABP4 on Cellular Metabolic Functions in HOCF Cells

**Figure 2:**
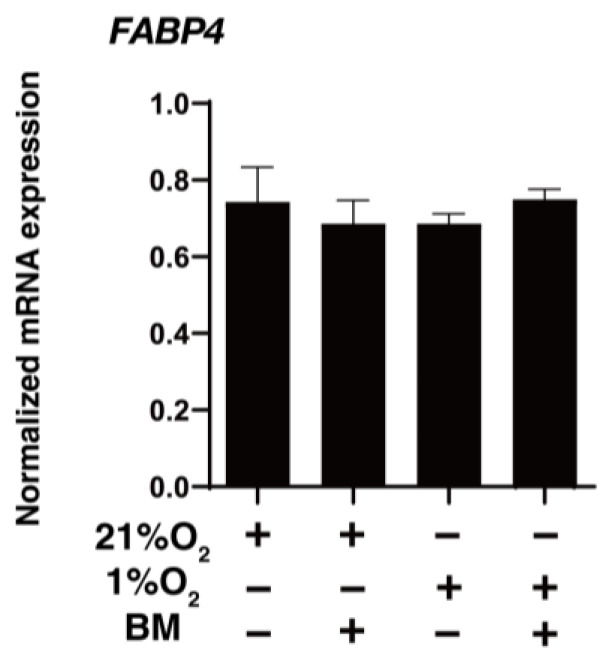
Effects of BMS309403 on mRNA expression of FABP4. Non-treated 2D HCOF cells or 10 μM BMS309403 (BM)-treated 2D HOCF cells cultured under normoxia conditions (21% O_2_) or hypoxic conditions (1% O_2_) were loaded for qPCR analysis of *FABP4*. Experiments were performed in duplicate (n = 3).

**Figure 3:**
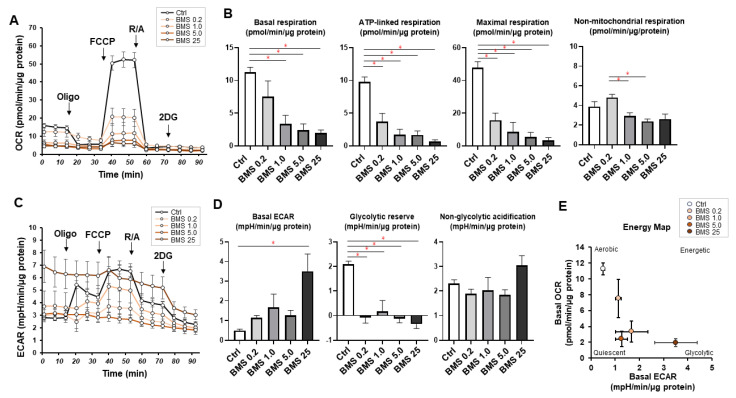
Effects of pharmacological FABP4 inhibition by BMS309403 on cellular metabolic functions. Two-dimensional cultured HCOF cells were not treated (Ctrl) or were treated with 0.2, 1.0, 5.0 or 25 μM BMS309403 (BMS), a specific inhibitor of FABP4, and each sample (n = 4–6) was loaded for real-time metabolic function analysis using a Seahorse XFe96 Bioanalyzer. (**A**) Measurements of oxygen consumption rates (OCRs). (**B**) Major indices of OCRs. (**C**) Measurements of extracellular acidification rates (ECARs). (**D**) Major indices of ECARs. (**E**) Energy map. * *p* < 0.05. Oligo: oligomycin. FCCP: carbonyl cyanide p-trifluoromethoxyphenylhydrazone. R/A: a mixture of rotenone/antimycin A. 2DG: 2-deoxyglucose.

**Figure 4:**
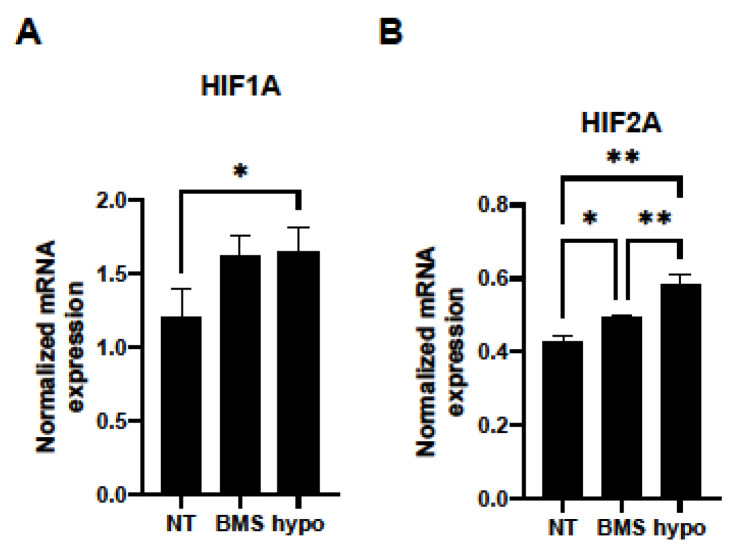
mRNA expressions of (**A**) HIF1a and (**B**) HIF2a. Non-treated 2D HCOF cells or 10 μM BMS309403-treated 2D HOCF cells cultured under normoxia conditions (21% O_2_) or non-treated 2D HOCF cells cultured under hypoxic conditions (1% O_2_) were subjected to qPCR analysis of *HIF1a* and *HIF2a*. Experiments were repeated twice (n = 3 each). * *p* < 0.05, ** *p* < 0.01.

BMS309403 is designed to interact with the fatty acid-binding pocket and is widely used as a potent and selective inhibitor for FABP4 [23]. To elucidate the physiological roles of FABP4 in HOCF cells using BMS309403, mitochondrial and glycolytic functions, as essential cellular functions, were examined by a Seahorse XFe96 Bioanalyzer. No effects of BMS309403 on the expression of FABP4 in HOCF cells were observed under either normoxia or hypoxic conditions, suggesting that BMS309403 indeed reacted with FABP4 at the protein level (Figure 2). As shown in Figure 3A,B, treatment with BMS309403 significantly decreased various indices of mitochondrial respiratory functions in HOCF cells, even with a low concentration of BMS309403. On the other hand, ECAR, at baseline reflecting basal glycolysis, showed an increased trend from the treatment with BMS309403 (Figure 3C,D). In addition, there was no increase in the glycolytic reserve, which reflects a compensatory increase in glycolysis in the presence of BMS309403 in HOCF cells (Figure 3C,D), suggesting that the increase in basal glycolysis is already saturated by the presence of BMS309403. Indeed, the energy map visualized a shift of the basal metabolism from mitochondrial respiration to glycolysis with the presence of BMS309403 (Figure 3E). These results suggest that pharmacological inhibition of FABP4 in HOCF cells shifts cellular metabolism from mitochondrial respiration to glycolysis. In support of these results, 10 μM BMS309403 or hypoxic conditions induced significant up-regulation of the mRNA expressions of *HIF1a* and *HIF2a* (Figure 4). These collective results suggested that FABP4 may be an indispensable survival factor for HOCF cells by regulating cellular homeostasis.

### 3.3. Functional Analysis of Differentially Expressed Genes That Were Modulated by the Pharmacological Inhibition of FABP4

**Figure 5:**
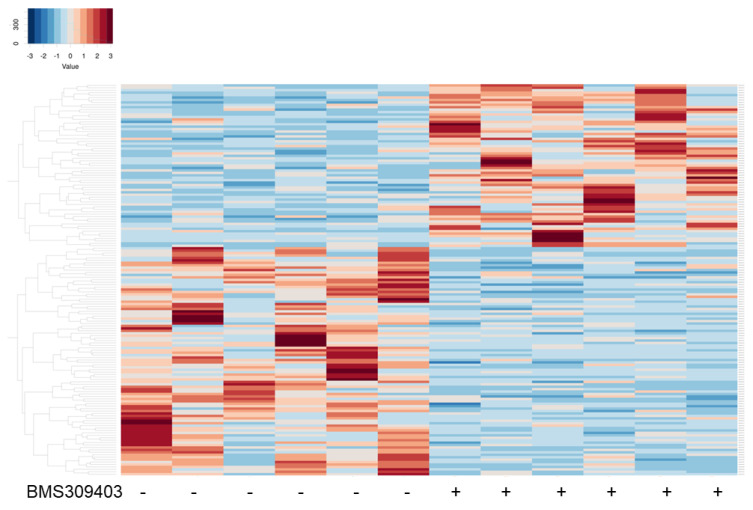
Heatmap for DEGs for BMS309403-untreated HOCF cells (NT 1-6) and BMS309403-treated HOCF cells (BMS 1-6). Two-dimensional cultured HOCF cells not treated with BMS309403 (NT, n = 6) at Day 6 and those treated with 5 μM BMS309403 (BMS, n = 6) were loaded for RNA sequencing analysis. Differentially expressed genes (DEGs) are shown by a hierarchical clustering heatmap. Colored bars represent either overexpressed (red) or underexpressed (blue) DEGs in NT cells compared with BMS cells.

**Figure 6:**
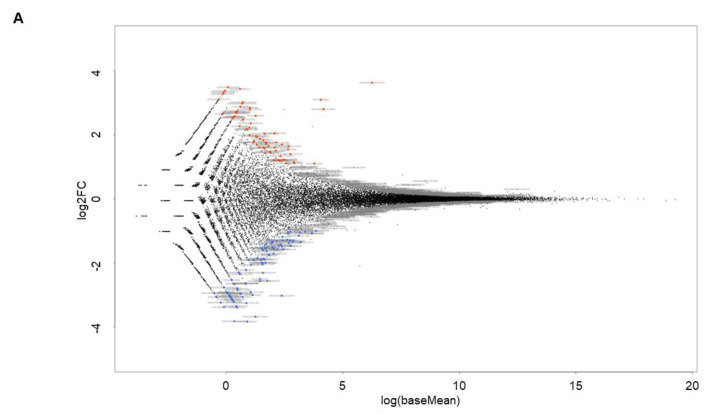

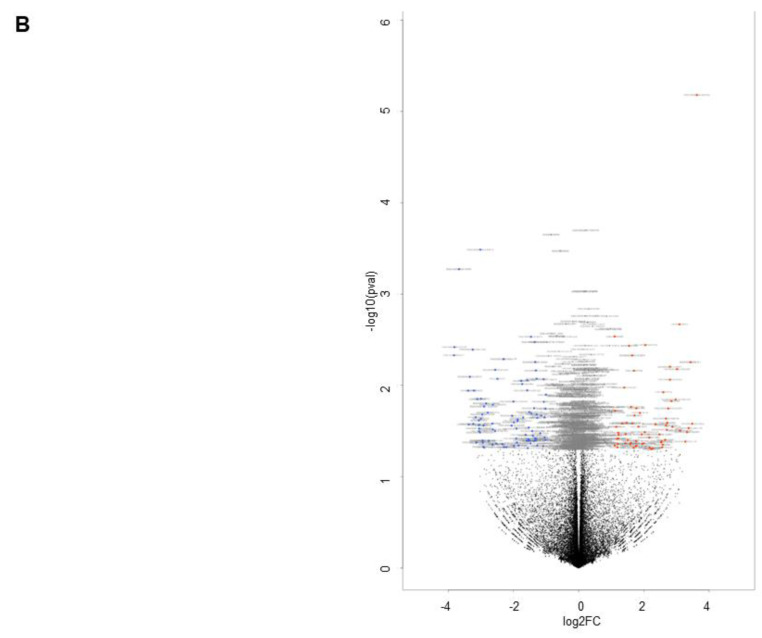
M-A plot (**A**) and volcano plot (**B**) for BMS309403-untreated HOCFs (NT 1-6) and BMS309403-treated HOCFs (BMS 1-6). Two-dimensional cultured HOCF cells not treated with BMS309403 (NT, n = 6) at Day 6 and those treated with 5 μM BMS309403 (BMS, n = 6) were loaded for RNA sequencing analysis. Differentially expressed genes (DEGs) are shown by an M–A plot (**A**) and a volcano plot (**B**). Colored points represent either overexpressed (red) or underexpressed (blue) DEGs in NT cells compared with BMS cells.

To further elucidate the kinds of molecular signaling that are related to FABP4 in HOCF cells, BMS309403-untreated HOCH cells (NT, n = 6) and BMS309403-treated HOCF cells (BMS, n = 6) were subjected to RNA sequencing analysis. As demonstrated in a heatmap (Figure 5) and an M-A (Figure 6A) and a volcano plot (Figure 6B), 67 markedly up-regulated and 94 markedly down-regulated DEGs were identified in BMS309403-untreated and -treated HOCH cells (a list of all of the DEGs is included in the Supplemental Excel File, Table S2). As the most prominent DEGs, the top 10 up-regulated and down-regulated molecules are shown in Table 1.

**Figure 7:**
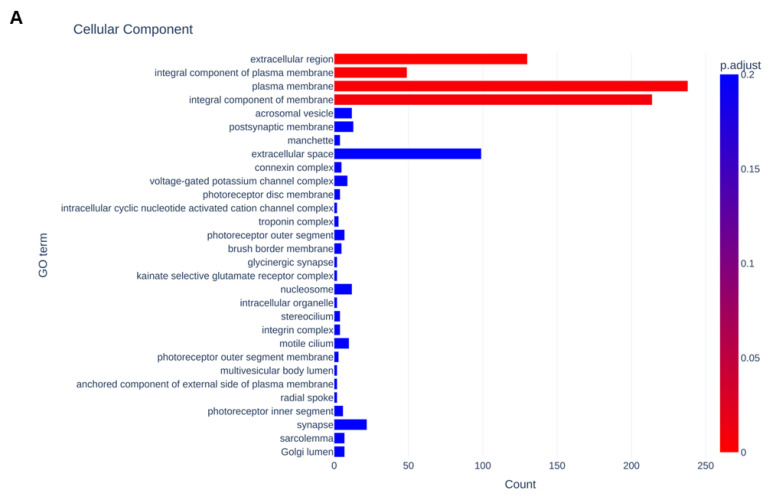

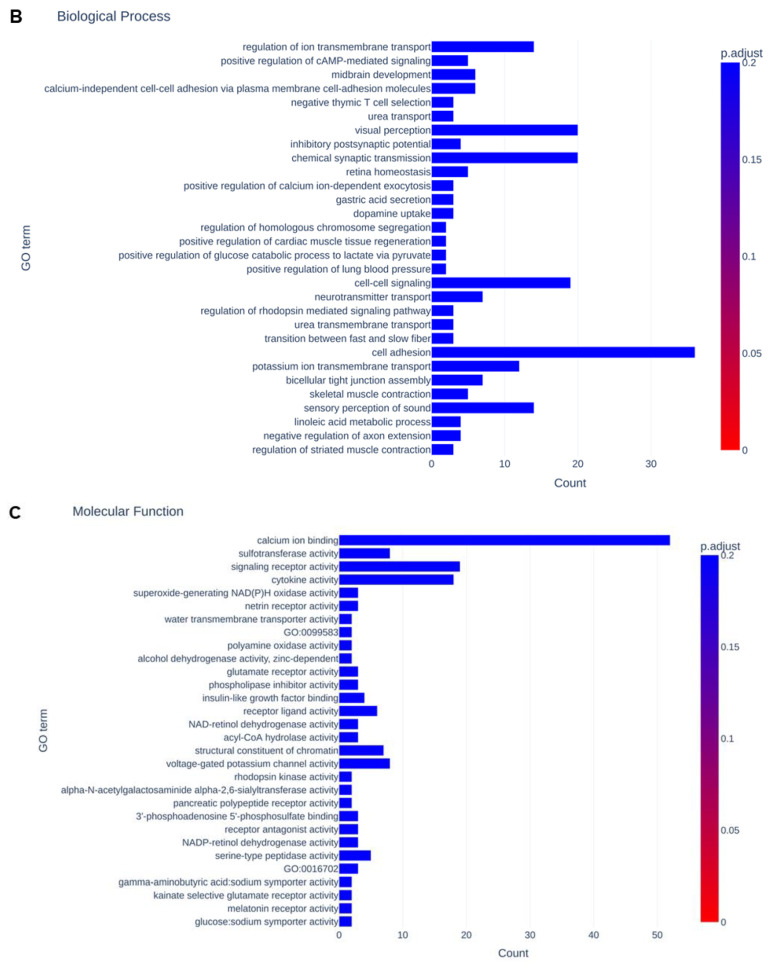
Results of GO enrichment analysis. (**A**) Cellular components, (**B**) biological processes and (**C**) molecular functions. Bar color represent *p*-values, and *x*-axis represents number of DEGs.

To estimate unidentified biological aspects of FABP4 in HOCF cells, GO enrichment analysis was performed. The detected DEGs were more abundantly categorized in GO terms related to (1) the plasma membrane, integral cellular component and extracellular component among cellular components (Figure 7A); (2) cell adhesion, visual perception, chemical synaptic transmission and cell–cell signaling among biological processes (Figure 7B); and (3) calcium ion binding among molecular functions (Figure 7C).

To assess additional biological aspects related to DEGs, IPA analysis was used. Based on the detected DEGs, estimations by IPA analysis were as follows: (A) the top five molecular and cellular functions (Table 2) were (1) cellular movement, (2) cell signaling, (3) post-translational modification, (4) cellular compromise and (5) molecular transport; (B) the top five canonical pathways (Table 3) were (1) rhodopsin-like receptors, (2) G-alpha (i) signaling events, (3) cAMP-response element-binding protein (CREB) signaling in neurons, (4) the S100 family signaling pathway and (5) visual phototransduction and adrenergic receptor signaling; and (C) the top five networks (Table 4) were (1) developmental disorder, (2) cell morphology, (3) cell-to-cell signaling and interaction, (4) cell death and survival and (5) neurological disease. In addition, the IPA upstream analysis suggested that NKX2-1 (thyroid transcription factor1), HOXA10 (homeobox A10), GATA2 (gata2 protein) and CEBPA were upstream regulators and that NKX2-1, SFRP1 (Secreted frizzled-related protein 1) and TREM2 (triggering receptor expressed on myeloid cells 2) were causal network master regulators. Indeed, the IPA findings showed that lipid-related factors ADIPOQ and CEBPA were estimated to be in the top five regulator effect networks (Table 5). Collectively, the results from RNA sequencing suggested that the selective FABP4 inhibitor BMS309403 affects some FABP4-related signaling to regulate the cellular homeostasis of non-adipocyte HOCF cells.

## 4. Discussion

Anatomically, the ocular choroid is in physical contact with the outer retina and supplies various nutrients, oxygen and biological factors via blood circulation [24]. Within the outer retina, RPE, a monolayer of polarized epithelial cells, is in direct contact with photoreceptor outer segments (OSs) and functions for photoreceptor survival and functions by daily phagocytosis of OS tips, recycling 11-cis retinal during phototransduction and maintaining the blood–retinal barrier [25]. In the current study, we found that (1) intraocularly present FABP4 originates from the ocular choroid, and (2) inhibition of FABP4 pivotally regulates essential cellular functions for survival, that is, mitochondrial and glycolytic functions, in HOCF cells. Furthermore, IPA analysis of RNA sequencing suggested that visual transduction-related roles were related to FABP4 in the HOCF cells in the top five canonical pathways. In our preceding study, we showed significant increases in both a- and b-waves of ERG amplitudes in Fabp4^−/−^ mice compared with those in WT mice [15]. We also found that positive immunoreactivities against FABP4 were detected in most of retinal segments except the photoreceptor outer segment (OS) [15]. Therefore, collectively, it was rationally speculated that FABP4 secreted from the ocular choroid may regulate not only the initial phototransduction by OS but also the following phototransduction by the mid-retina, which are the origins of the a-wave and b-wave of ERG, respectively. If this speculation is correct, the retinal choroid may be included in the sensory retina. In fact, various studies have also suggested the concept of an “RPE/choroid complex” [26,27] and that this complex is involved in various ocular diseases, such as AMD [28] and myopia [29]. More interestingly, in the current study, we also used a Seahorse cellular metabolic analysis and found that inhibition of FABP4 by BMS309403 induced so-called pseudohypoxic states. If this is the case, such pseudohypoxia may lead to neovascularization, as was suggested by using an AMD model [30] and malignant tumors [31,32], and this scenario may strongly support our recent proposal that FABP4 is an independent key pathogenic factor for retinal vascular diseases (RVDs) [15] such as DR [9] and RVO [10] in addition to intraocular physiology.

As for the molecular mechanisms underlying FABP4 suppression-induced pseudohypoxia in HOCF cells, in addition to HIF mechanisms, calcium-related signaling may be involved, as suggested by a previous study [33]. In support of this idea, IPA analysis indicated that (1) cell signaling was included in the top five molecular and cellular functions, (2) the calcium-binding protein S100-related signaling pathway was included in the top five canonical pathways and (3) upstream regulators NKX2-1 [34], HOXA10 [35], GATA2 [36] and CEBPA [37] and causal network master regulators NKX2-1 [34], TRPM2 [38] and SFRP1 [39] are known to regulate Ca^2+^ signaling and/or mitochondrial functions.

However, as limitations of this study, the following issues need to be investigated. Firstly, although previous studies have suggested that FABPs are required for development of the retina and BRB in zebrafish [40,41] and chickens [42,43] and a recent study using a Drosophila ninaEG69D mutant demonstrated that FABP is required for light-induced Rh1 degradation and photoreceptor survival [44], suggesting that FABPs are pivotally involved in intraocular homeostasis, the precise roles of intraocularly present FABPs in addition to FABP4 remain to be elucidated. Secondly, although FAs, substrates of FABPs, have been well characterized in the pathophysiology of photoreceptors which require high energy demand [45,46], there is little information in terms of the roles of FAs in other intraocular cell types. In our recent study, we found synergistic elevations of vitreous levels of FABP4, FABP5 and FAs in patients with RVO [15], strongly suggesting that FAs are exclusively required for the biological activities of FABPs, although details of those have not been identified yet. Thirdly, the fact that lipid metabolism regulators, FABPs, were indeed involved in intraocular homeostasis despite no adipocytes being present suggests that other lipid metabolism regulators, such as PPARα and PPARγ, are involved in intraocular pathophysiology [47]. Fourthly, as for the rationale to use BMS309403 as a specific FABP4 inhibitor, additional experimental proof would be required to make sure that BMS309403 indeed inhibits FABP4 but not other factors in non-adipocyte HOCF cells. However, several studies have shown that BMS309403 is in the first line of selective and effective FABP4 inhibitors among hundreds of other synthesized inhibitors, including derivatives of niacin, quinoxaline, aryl-quinoline, bicyclic pyridine, urea, aromatic compounds and other compounds [23,48,49]. In addition, the present RNA sequencing analysis estimated adipocyte-related factors ADIPOQ and CEBPA as the regulators among top five regulator effect networks, suggesting that BMS309403 indeed inhibits FABP4 in HOCF cells. Fifthly, in this study, only four different cells originated from intraocular tissues were used, and other types of intraocularly originated cells may also express FABP4. In addition, the biological natures of these commercially available cells may be different from those of in vivo native and matured conditions. Therefore, investigations to solve these unidentified issues in conjugation with additional investigation to find out new key molecules among obtained DEGs will be our next projects.

In conclusion, FABP4-related lipid metabolism regulation is a critical mechanism for the maintenance of intraocular homeostasis, and this may provide a suitable model for understanding the roles of FABP4 in non-adipose tissues.

## Figures and Tables

**Table 1:** Top 10 up-regulated and down-regulated DEGs between BMS309403-untreated HOCFs and BMS309403-treated HOCFs.

Up-Regulation		Down-Regulation	
Molecules	*p*-Value	Molecules	*p*-Value
KIF1A	6.301	CHRNA1	−5.838
CDHR1	6.156	TNFSF18	−5.603
CSTA	5.410	IDO1	−5.018
LCN1	5.271	LOC105377155	−4.997
HSPA6	4.990	LOC102724434	−4.661
LINC01705	4.837	ENSG00000289492	−4.658
PIP5K1B	4.719	CFI	−4.610
LINC02568	4.679	AKAP13-AS1	−4.605
ZBBX	4.607	MYO16	−4.555
AGA-DT	4.593	RP11_885L141	−4.502

**Table 2:** Top 5 molecular and cellular functions.

Name	*p*-Value of Range
Cellular Movement	1.58 × 10^−6^–1.98 × 10^−21^
Cellular Development	1.47 × 10^−6^–4.12 × 10^−19^
Cellular Function and Maintenance	5.00 × 10^−7^–4.12 × 10^−19^
Cellular Growth and Proliferation	1.24 × 10^−6^–4.12 × 10^−19^
Cell-to-Cell Signaling and Interaction	1.58 × 10^−6^–6.61 × 10^−16^

**Table 3:** Top 5 canonical pathways.

Name	*p*-Value
rhodopsin-like receptors	2.34 × 10^−8^
G-alpha (i) signaling events	3.08 × 10^−6^
cAMP-response element-binding protein (CREB) signaling in neurons	6.25 × 10^−6^
S100 family signaling pathway	7.71 × 10^−6^
visual phototransduction and adrenergic receptor signaling	1.51 × 10^−5^

**Table 4:** Top 5 networks.

Name	Score
developmental disorder	43
cell morphology	41
cell-to-cell signaling and interaction	41
cell death and survival	37
neurological disease	34

**Table 5:** Top five regulator effect networks.

Regulators	Disease and Function	Consistency Score
**ADIPOQ**, IL25, RNF31	acute disease, immune response of leukocytes	7.542
CASP4, **CEBPA**, CLEC11A, RARB	acute disease, hypertensive reaction	2.296
**ADIPOQ**	polarization of myeloid cells	−6
**ADIPOQ**	polarization of phagocytes	−6
RARB	secretion of neurotransmitter	−8.083

## Data Availability

The data that support the findings of this study are available from the corresponding author upon reasonable request.

## Supplementary Materials

The following supporting information can be downloaded at https://www.mdpi.com/article/10.3390/bioengineering11060584/s1: Table S1: Primers used in the present study; Table S2: A list of all of the up-regulated and down-regulated DEGs.
